# Cultural sustainability through kinship networks: A quantitative analysis of marriage strategies in Qing China’s literary lineages (1644–1911)

**DOI:** 10.1371/journal.pone.0359981

**Published:** 2026-10-06

**Authors:** Yifan Gu

**Affiliations:** School of Humanities and Arts, China University of Mining and Technology, Xuzhou, Jiangsu, China; Bahir Dar University, ETHIOPIA

## Abstract

This study examines how marriage-network position was associated with recorded literary visibility among Qing literary families. Using Xu Yanping’s Genealogical Networks of Literary Lineages in the Qing Dynasty (2010), we reconstructed a reproducible surname–location-level marriage network from an OCR-based machine-readable corpus. After OCR correction, node normalization, and removal of self-loops, the final analytical dataset contained 5,197 parseable marriage records, 657 focal literary-family nodes, 2,704 total family nodes including non-focal marriage partners, and 3,541 distinct weighted family-pair edges. We calculated weighted degree, unweighted degree, betweenness centrality, eigenvector centrality, community structure, and global network indicators. To avoid overclaiming from incomplete examination-degree information in the OCR corpus, we used a conservative Literary Capital Proxy (LCP) based on observable literary-output clues and confirmed female-writer clues. Regression models with province-clustered robust standard errors show that unweighted degree, measured as the number of distinct marital partner families, is positively associated with LCP after controlling for record exposure and regional differences. Weighted degree shows a weaker and less consistent association. Betweenness centrality is negatively associated with LCP in the exposure-adjusted models, while eigenvector centrality is not statistically significant. These findings suggest that the breadth of direct marital connections was more consistently related to recorded literary visibility than repeated marriage volume, brokerage position, or connection to already central families. The results should be interpreted as associational rather than causal, because marriage ties, literary visibility, wealth, officeholding, education, regional culture, and archival survival may have shaped one another. The study contributes a transparent network-based framework for examining kinship, marriage, and cultural transmission in Qing China.

## 1. Introduction

### 1.1. Family, marriage, and cultural reproduction in imperial China

Cultural sustainability concerns how communities preserve, transmit, and reinterpret cultural knowledge, practices, and identities across generations. In simple terms, it asks how cultural memory continues over time and what social structures help that memory survive. In historical societies, such transmission did not depend only on formal institutions. It was also embedded in family organization, marriage alliances, education, textual production, and the preservation of genealogical and literary records. Qing China provides a useful context for examining these issues because literary reputation, examination achievement, family memory, and marriage strategies were closely connected within elite and gentry society.

Recent scholarship has increasingly recognized the role of kinship networks in shaping the intellectual and social landscape of late imperial China [1,2]. Conventional historiography has emphasized patrilineal descent, private tutoring, lineage organization, and the civil examination system as major mechanisms of cultural reproduction. However, marriage also linked families across patrilines and created channels through which education, reputation, manuscripts, poetic practice, and women’s literary memory could circulate [3–5]. Marriages among scholar-official and literary families were therefore not merely private domestic events. They were also relational connections that could affect how families were remembered, documented, and positioned within broader cultural networks.

The concept of cultural capital, as developed by Bourdieu [6], provides a useful theoretical language for understanding these dynamics. In the Chinese context, cultural capital could take institutionalized form through examination degrees, objectified form through books, manuscripts, and collected writings, and embodied form through scholarly training, literary practice, and family habitus [7]. Literary families were not only groups of individual writers. They were also social units whose reputation depended on intergenerational education, textual preservation, marriage connections, and recognition by other elite families.

In this study, cultural sustainability is used in a limited historical sense. It refers to the preservation and circulation of recorded literary visibility across families and generations, rather than to a direct modern policy framework. Marriage networks are examined as one relational structure associated with such visibility. The study does not assume that marriage ties caused literary achievement or that kinship networks automatically produced resilience. Instead, it investigates whether measurable positions in the marriage network were associated with observable literary-output and female-authorship clues in the reconstructed source corpus.

Previous scholarship relevant to this study can be grouped into three broad strands. The first strand examines elite lineages, examination success, and cultural reproduction in late imperial China, emphasizing patrilineal descent, education, officeholding, and family reputation. The second strand focuses on marriage alliances and women’s literary culture, showing that marital ties could connect natal and marital families and influence the preservation of women’s writings. The third strand comes from digital humanities and historical network analysis, which uses structured data and network measures to examine social relations that are difficult to observe through individual biographies alone. Building on these strands, the present study contributes a quantitative network analysis of how marriage breadth and centrality were associated with recorded literary visibility among Qing literary lineages.

Despite the richness of genealogical and literary records, large-scale quantitative analysis of Qing literary intermarriage remains limited. Most existing studies are qualitative, regionally focused, or centered on a small number of prominent lineages. Pan Guangdan’s study of Jiaxing lineages mapped marriage relations through genealogical diagrams, while later historical studies examined how landholding, officeholding, and marriage alliances shaped elite family continuity [3,8]. Xu Yanping’s comprehensive source work provides a valuable foundation for extending this line of inquiry, but its materials require careful parsing, normalization, and transparent computational reconstruction before they can be used for network analysis [5].

### 1.2. Digital humanities and the analysis of historical kinship networks

The digital turn in Sinology has made it increasingly possible to examine historical social relations at larger scales. Databases and tools such as the China Biographical Database, MARKUS, and related digital humanities platforms allow scholars to process biographical, textual, and geographical materials in more systematic ways [9,10]. Recent studies have shown how computational methods can transform unstructured or semi-structured historical texts into analyzable data. For example, digital approaches have been used to classify military roles in the *Ming Shilu* and to analyze historical meteorological records in spatial and temporal contexts [11,12]. These studies show that computational analysis can complement close reading when the procedures for data extraction and interpretation are made explicit.

Historical kinship and marriage networks are especially suitable for network analysis because they involve relational data. Previous studies of European elites, such as the Medici family in Florence, Dutch regents, British nobility, and French magistrates, have demonstrated that marriage networks can be associated with political, economic, and social position [13–16]. These studies do not imply that all marriage-network centrality has the same meaning across contexts. Rather, they show that network measures can help identify patterns of alliance, brokerage, clustering, and social reach that are difficult to observe from individual biographies alone.

This study applies a similar relational approach to Qing literary families. The goal is not to equate Qing literary lineages with European political elites, nor to assume that marriage centrality directly produced cultural power. Instead, the study asks how different forms of marriage-network position were associated with recorded literary visibility in a non-European historical context. This requires distinguishing between different centrality measures. Weighted degree captures repeated marriage volume, unweighted degree captures the number of distinct partner families, betweenness captures brokerage position, and eigenvector centrality captures connection to highly connected families. Treating these measures separately allows the analysis to avoid the ambiguous use of “degree” and to test which network features are most closely associated with the Literary Capital Proxy.

The analysis also responds to the methodological challenges of working with OCR-based historical sources. Printed genealogical and literary records contain irregular headings, repeated family names, incomplete locality markers, and inconsistent documentation of women’s names and authored works. For this reason, the study adopts a conservative surname–location aggregation strategy and interprets the resulting network as a reproducible analytical reconstruction rather than a perfect reproduction of every genealogical distinction in the printed source. This approach prioritizes transparency, replicability, and cautious interpretation.

### 1.3. Research questions and approach

The objective of this study is to quantitatively examine whether, and in what ways, marriage-network position was associated with the maintenance and circulation of recorded literary visibility among Qing literary families. More specifically, this study asks how marriage-network structure was associated with recorded literary visibility among Qing literary families. Rather than treating marriage networks as direct causal mechanisms of cultural capital accumulation, the revised analysis frames them as relational structures through which literary visibility, family reputation, and female authorship may have been recorded, circulated, and preserved.

The study addresses three research questions:

(1) What are the global structural features of the Qing literary marriage network after reproducible OCR correction, surname–location normalization, and weighted edge construction?
(2) Which focal literary-family nodes occupy central positions when weighted degree, unweighted degree, betweenness centrality, and eigenvector centrality are distinguished?
(3) How is family network position associated with the Literary Capital Proxy, a conservative measure of recorded literary visibility based on observable literary-output clues and confirmed female-writer clues?

To answer these questions, we reconstruct a weighted, undirected marriage network from Xu Yanping’s *Genealogical Networks of Literary Lineages in the Qing Dynasty*. The printed source records 5,289 marriage entries involving 766 literary-family entries. For reproducible computational analysis, the OCR-based corpus was parsed and normalized at the surname–location level. The final analytical dataset contains 5,197 parseable marriage records, 657 focal literary-family nodes, 2,704 total family nodes including non-focal marriage partners, and 3,541 distinct weighted family-pair edges.

The analysis proceeds in four steps. First, we construct a family-level marriage network and report global topological indicators, including the largest connected component, clustering coefficient, modularity, and community structure. Second, we calculate weighted degree, unweighted degree, betweenness centrality, and eigenvector centrality to distinguish different forms of network position. Third, we construct the Literary Capital Proxy from observable textual clues in the parsed corpus and examine whether network measures are associated with this proxy among focal literary-family nodes. Fourth, we conduct robustness and diagnostic analyses, including alternative LCP specifications, province-clustered robust standard errors, a record-exposure proxy, variance inflation checks, residual diagnostics, discordant-case analysis, and a node-removal structural robustness simulation.

The study therefore tests a limited associational claim: whether broader or more central marital connections are associated with recorded literary visibility in the available source corpus. Because the reconstructed data are observational and lack sufficient temporal precision, the analysis does not claim that marriage ties caused literary capital accumulation or historical adaptation to specific shocks.

## 2. Data and methodology

### 2.1. Source corpus and analytical scope

The primary source for this study is Xu Yanping’s *Genealogical Networks of Literary Lineages in the Qing Dynasty* (2010), the most comprehensive published reference work on marital relations among Qing literary families. The source work records 5,289 marriage entries involving 766 literary-family entries. These records were compiled from Qing examination records, genealogies, local gazetteers, collected works, women’s literary catalogues, epitaphs, and other historical materials.

For the purpose of reproducible computational analysis, the present study distinguishes between the bibliographic scope of Xu’s source work and the analytical dataset constructed from the OCR-based machine-readable corpus. Because some entries in the OCR corpus contained irregular punctuation, damaged marriage markers, repeated lineage headings, or homonymous families from the same locality, we adopted a conservative surname–location aggregation rule. Under this rule, a family node is defined by surname and recorded locality, such as Zhang family of Tongcheng or Chen family of Haining. When multiple entries shared the same surname–location identifier and could not be reliably separated from the OCR text alone, they were aggregated into a single analytical family unit.

After OCR correction, text parsing, node normalization, removal of self-loops, and aggregation at the surname–location level, the final reproducible dataset used in this study contains 5,197 parseable marriage records. The resulting network includes 2,704 total family nodes, including both focal literary-family nodes and non-focal marriage partners. Among these, 657 nodes are focal literary-family nodes identified from family headings in the source corpus. The network contains 3,541 distinct weighted family-pair edges, of which 1,271 connect two focal literary-family nodes and 2,270 connect a focal literary family to a non-focal or externally recorded marriage partner. The sum of edge weights is 5,173 after excluding 24 self-loops.

This analytical scope is intentionally conservative. It does not claim to reproduce every genealogical distinction in Xu’s printed source, especially where homonymous lineages from the same locality could not be separated with sufficient confidence. Instead, it provides a transparent and reproducible network dataset suitable for statistical analysis and public data sharing.

### 2.2. Data parsing and node construction

The raw text was processed using a rule-based parsing workflow implemented in R. The workflow identified marriage markers such as “娶” and “适”, extracted the family heading under which each entry was recorded, and identified the marriage partner’s surname and locality from the first locality marker following the marriage verb. Common OCR variants of marriage markers and brackets were normalized before parsing.

Family nodes were standardized at the surname–location level. For example, entries associated with “张氏（安徽桐城）” were assigned to the node “Zhang family of Tongcheng, Anhui”. This rule reduces ambiguity caused by personal names, generational relationship terms, and repeated entries. It also avoids the erroneous creation of personal-name nodes, such as treating “Li Hongzhang” or “Zhang Peilun” as separate family units.

The edge list was constructed as an undirected weighted network. Each edge represents at least one recorded marital connection between two family nodes. If multiple marriages were recorded between the same two family nodes, the edge weight equals the number of recorded marriage ties. Self-loops, defined as marriages recorded within the same surname–location unit, were excluded from the main network analysis but retained in the data-processing log.

The cleaned dataset includes three main files: a parsed entry-level file, a weighted edge list, and a node-level attribute file. These files, together with the R scripts used for parsing, network construction, and statistical analysis, are made available for replication.

## 3. Methods

### 3.1. Network measures

This study distinguishes between unweighted degree and weighted degree. Unweighted degree refers to the number of distinct family nodes with which a given family formed marital ties. Weighted degree, also referred to as marriage strength, refers to the total number of recorded marriage ties attached to a family after edge weights are summed.

Betweenness centrality was calculated to measure the extent to which a family occupied a bridging position between otherwise separated parts of the network. Because edge weights in this study represent tie strength rather than distance, inverse edge weight was used as the path distance when computing betweenness centrality. This avoids treating repeated marital connections as longer paths. Eigenvector centrality was calculated to capture whether a family was connected to other highly connected families.

Community structure was identified using the Louvain algorithm [17]. The largest connected component, average path length, diameter, global clustering coefficient, and modularity were calculated to describe the overall topology of the network. These indicators are used to describe structural properties of the marriage network, rather than to make direct causal claims about historical adaptation to specific political shocks.

### 3.2. Literary Capital Proxy

The original version of this study proposed a composite Literary Capital Score based on examination success, literary output, and female authorship. During revision, we adopted a more conservative measure, the Literary Capital Proxy (LCP), because complete examination-degree counts could not be fully reconstructed from the OCR-based reproducible corpus.

The LCP is based on two observable components that can be extracted from the parsed source text: literary-output clues and confirmed female-writer clues. Literary-output clues refer to records in which the source text explicitly notes the existence of authored works, usually marked by phrases such as “has writings” or by the inclusion of titles in book brackets. Confirmed female-writer clues refer to cases where the parser could identify the family associated with a woman writer in the marriage entry.

The main LCP specification assigns a weight of 0.6 to literary-output clues and 0.4 to confirmed female-writer clues after log transformation and min–max normalization. To address concerns about the weighting scheme, we also estimated alternative specifications, including equal weighting, a literary-output-heavy specification, a female-authorship-heavy specification, a binary female-authorship specification, and a PCA-derived proxy when the data structure allowed. These sensitivity analyses were used to assess whether the association between network position and literary visibility depended on a single weighting choice.

Because the LCP is based on observable textual clues rather than a complete reconstruction of all literary and examination achievements, it should be interpreted as a conservative proxy for recorded literary visibility within the source corpus, not as a complete measure of a family’s total cultural capital.

### 3.3. Regression models and robustness checks

The main statistical analysis examines whether network position is associated with the Literary Capital Proxy among focal literary-family nodes. The dependent variable is the LCP. The main predictors are weighted degree, unweighted degree, betweenness centrality, and eigenvector centrality. All continuous network variables were standardized before regression to improve comparability.

To address the concern that larger or more frequently recorded families may have more opportunities to form marriage ties and to appear in literary records, the models include a record-exposure proxy measured as the log-transformed weighted degree. This variable is not interpreted as a direct measure of biological family size; rather, it serves as a conservative control for the amount of recorded relational exposure in the source corpus.

Regional differences were controlled using core-region categories, distinguishing Jiangsu, Zhejiang, Anhui, and other regions. Because families within the same province may share cultural, economic, and archival conditions, standard errors were clustered at the province level. This adjustment reduces the risk that statistical significance is inflated by regional dependence.

The revised models are interpreted as associational rather than causal. The cross-sectional structure of the reconstructed network does not allow us to determine whether marital centrality produced literary visibility, whether more literary families attracted more marriage ties, or whether both were shaped by omitted historical factors such as wealth, officeholding, education, and archival survival. The results therefore identify robust associations between network position and recorded literary visibility, not causal effects.

Additional robustness checks include alternative LCP definitions, comparison of weighted and unweighted degree models, diagnostic tests for heteroskedasticity and influential observations, variance inflation factor checks, and a discordant-case analysis identifying families with high centrality but low LCP and families with low centrality but high LCP.

### 3.4. Ethics statement

This study used published historical and genealogical records and did not involve living human participants, patients, interviews, surveys, medical records, archived biological samples, or identifiable private information. Therefore, institutional ethics approval and informed consent were not required.

## 4. Results

### 4.1. Revised network scope and global topology

Table 1 reports the basic attributes of the revised analytical network. After OCR correction, parsing, surname–location normalization, and removal of self-loops, the reproducible dataset contains 5,197 parseable marriage records. A total of 24 self-loops were excluded from the main network analysis. The final network includes 2,704 family nodes, including both focal literary-family nodes and non-focal marriage partners. Among these, 657 nodes are focal literary-family nodes identified from family headings in the source corpus.

**Table 1:** Revised network attributes.

Indicator	Value
Parseable marriage records	5,197
Self-loops excluded	24
Total family nodes, including non-focal marriage partners	2,704
Focal literary-family nodes	657
Distinct weighted family-pair edges	3,541
Within-focal-network edges	1,271
External/non-focal-family edges	2,270
Sum of edge weights	5,173
Giant component nodes	2,246
Giant component edges	3,288
Average path length, giant component	3.50
Diameter, giant component	12.08
Global clustering coefficient	0.024
Louvain modularity	0.748

The network contains 3,541 distinct weighted family-pair edges. Of these, 1,271 edges connect two focal literary-family nodes, while 2,270 edges connect focal literary families with non-focal or externally recorded marriage partners. The sum of edge weights is 5,173, indicating that multiple marriage records often connected the same family pairs.

The largest connected component contains 2,246 nodes and 3,288 edges, showing that most families were embedded in a large connected structure rather than appearing as isolated dyads. The average path length of the giant component is 3.50, and the weighted network diameter is 12.08. The global clustering coefficient is 0.024, while Louvain modularity is 0.748. These indicators suggest that the Qing literary marriage network was not a uniformly dense network. Rather, it was a large, modular network composed of regionally and genealogically clustered groups connected through selected inter-family ties.

### 4.2. Central families and the distinction between weighted and unweighted degree

Table 2 lists the top focal literary-family nodes ranked by weighted degree, that is, the total number of recorded marriage ties attached to each family. The results confirm the centrality of several well-known Qing literary lineages, especially families from Tongcheng, Haining, Wujin, Yixing, Qufu, Qiantang, and Wuxian.

**Table 2:** Top focal literary-family nodes ranked by weighted degree.

Family node	Province	W-degree	U-degree	Betweenness	Eigenvector	Literary clues	Female clues	LCP
Zhang family, Tongcheng	Anhui	309	57	0.042	0.984	9	9	0.749
Yao family, Tongcheng	Anhui	307	48	0.073	1.000	9	9	0.749
Chen family, Haining	Zhejiang	246	126	0.281	0.002	21	20	1.000
Fang family, Tongcheng	Anhui	194	66	0.153	0.477	10	10	0.780
Zhuang family, Wujin	Jiangsu	169	72	0.157	0.016	2	2	0.358
Ren family, Yixing	Jiangsu	114	44	0.052	0.001	4	4	0.524
Kong family, Qufu	Shandong	109	54	0.232	0.022	0	0	0.000
Xu family, Qiantang	Zhejiang	100	59	0.056	0.002	4	3	0.495
Pan family, Wuxian	Jiangsu	89	39	0.049	0.003	0	0	0.000
Wang family, Qiantang	Zhejiang	88	44	0.071	0.000	12	12	0.835

Notes: W-degree refers to weighted degree, defined as the total number of recorded marriage ties attached to a family. U-degree refers to unweighted degree, defined as the number of distinct partner families. Literary clues refer to observable literary-output clues identified in the parsed source corpus. Female clues refer to confirmed female-writer clues. LCP refers to the Literary Capital Proxy.

The Zhang family of Tongcheng, Anhui has the highest weighted degree (309), followed closely by the Yao family of Tongcheng (307), the Chen family of Haining (246), the Fang family of Tongcheng (194), and the Zhuang family of Wujin (169). These families occupy prominent positions in the marriage network and represent dense marriage accumulation within and beyond the Jiangnan literary core.

The distinction between weighted and unweighted degree is important. For example, the Zhang family of Tongcheng has a weighted degree of 309 but an unweighted degree of 57, indicating repeated marital connections with some partner families. By contrast, the Chen family of Haining has a weighted degree of 246 but an unweighted degree of 126, suggesting a broader range of distinct marital partners. This difference shows why the revised analysis reports both indicators rather than treating “degree” as a single measure.

The Chen family of Haining has the highest Literary Capital Proxy score among the top-ranked families (LCP = 1.000), with 21 literary-output clues and 20 confirmed female-writer clues. Several other high-centrality families also show high LCP scores, including the Wang family of Qiantang, the Fang family of Tongcheng, the Qian family of Xiushui, the Zhang family of Tongcheng, and the Yao family of Tongcheng. However, not all central families have high LCP scores. For example, the Kong family of Qufu and the Pan family of Wuxian have high marriage centrality but no confirmed literary-output or female-writer clues in the reproducible parsed corpus. This pattern supports the need for the discordant-case analysis reported below.

### 4.3. Network position and Literary Capital Proxy

Table 3 reports OLS regression models estimating the association between network position and the Literary Capital Proxy among focal literary-family nodes. All continuous predictors are standardized, and standard errors are clustered at the province level. The results should be interpreted as associations rather than causal effects.

**Table 3:** Revised regression models predicting Literary Capital Proxy.

Predictor	Model 1	Model 2	Model 3	Model 4
Weighted degree	0.039†	0.085*	—	—
Unweighted degree	—	—	0.083**	0.086**
Betweenness	—	−0.027†	−0.039*	−0.040*
Eigenvector centrality	—	−0.033	0.003	0.003
Record-exposure proxy	—	—	0.004	—
Zhejiang	0.034***	0.033**	0.022***	0.021**
Anhui	−0.058*	−0.029	−0.024	−0.024
Other regions	−0.060*	−0.049†	−0.048†	−0.048†
N	655	655	655	655
R²	0.152	0.174	0.228	0.227
Adjusted R²	0.147	0.166	0.219	0.220

Notes: Coefficients are standardized for continuous network variables. Standard errors are clustered at the province level. Model 1 includes weighted degree and core-region controls. Model 2 adds betweenness and eigenvector centrality. Model 3 is the exposure-adjusted model including unweighted degree and the record-exposure proxy. Model 4 uses unweighted degree without the exposure proxy. † p < 0.10, * p < 0.05, ** p < 0.01, *** p < 0.001.

Model 1 includes weighted degree and core-region controls. The coefficient for weighted degree is positive but only marginally significant (β = 0.039, p = 0.058). Model 2 adds betweenness and eigenvector centrality. In this specification, weighted degree becomes positive and statistically significant (β = 0.085, p = 0.039), while betweenness is negative but not significant at the 0.05 level (β = −0.027, p = 0.076). Eigenvector centrality is not significant.

Model 3 is the preferred exposure-adjusted model. It replaces weighted degree with unweighted degree and includes a record-exposure proxy to account for the possibility that more frequently recorded families had more opportunities to appear in both marriage and literary records. In this model, unweighted degree is positively and significantly associated with LCP (β = 0.083, p = 0.001). This finding suggests that families connected to a larger number of distinct marital partners tended to have higher recorded literary visibility.

Betweenness centrality is negatively associated with LCP in Model 3 (β = −0.039, p = 0.014). This result does not support the original expectation that brokerage position would necessarily correspond to higher literary visibility. One interpretation is that families occupying bridging positions may have connected otherwise separate marriage clusters without necessarily having more recorded literary-output or female-authorship clues in the source corpus. Eigenvector centrality remains non-significant (β = 0.003, p = 0.791), indicating that being connected to highly central families does not independently predict LCP once distinct partner range and regional factors are considered.

Model 4, which uses unweighted degree without the exposure proxy, produces substantively similar results. Unweighted degree remains positive and significant (β = 0.086, p = 0.004), betweenness remains negative and significant (β = −0.040, p = 0.017), and eigenvector centrality remains non-significant. These results indicate that the most stable association is between the breadth of distinct marriage partners and recorded literary visibility.

### 4.4. Sensitivity analyses, diagnostics, and discordant cases

The sensitivity analyses show that the positive association between unweighted degree and LCP is robust across alternative LCP definitions, including equal weighting, literary-output-heavy weighting, female-authorship-heavy weighting, and specifications using binary female authorship. Across these models, unweighted degree remains positive and statistically significant in the exposure-adjusted and unweighted-degree models. This indicates that the association between distinct marriage-partner range and recorded literary visibility is not dependent on a single weighting scheme. A post hoc sensitivity power analysis was conducted for the main exposure-adjusted model. With N = 655 and the included predictors, the model had 80% power at α = 0.05 to detect a small incremental effect of approximately f^2^ = 0.012, equivalent to a partial R^2^ of about 0.012 for a focal predictor. This result is reported as a sensitivity assessment rather than as evidence of causal identification.

The diagnostic tests suggest that robust standard errors are necessary. The Breusch–Pagan test indicates heteroskedasticity in the main model (p < 0.001), supporting the use of cluster-robust standard errors. The transformed GVIF values do not indicate severe multicollinearity. The largest transformed GVIF is 2.98 for unweighted degree, while the values for betweenness, eigenvector centrality, record exposure, and core region are all below 2.20. Cook’s distance identifies a small number of influential cases, but the mean Cook’s distance is low, suggesting that the main pattern is not driven by a large number of extreme observations.

The discordant-case analysis further clarifies the relationship between marital centrality and recorded literary visibility. The analysis identifies 62 high-centrality/high-LCP cases, 51 high-centrality/low-LCP cases, and 26 low-centrality/high-LCP cases. The presence of high-centrality/low-LCP cases confirms that marital centrality is not mechanically equivalent to literary visibility. Conversely, the low-centrality/high-LCP cases show that some families had substantial recorded literary visibility despite occupying less central positions in the marriage network. These cases support a more cautious interpretation: marital network breadth is associated with literary visibility, but it does not fully determine it.

Finally, the node-removal simulation is used only as a structural robustness analysis. It compares the effect of removing high-weighted-degree families with random node removal. This analysis describes the network’s topological vulnerability to the removal of highly connected families (Fig 1). The targeted removal of high-weighted-degree families reduces the largest connected component more rapidly than random removal, indicating that the network’s connectivity was structurally dependent on a limited number of highly connected family nodes.

**Fig 1:**
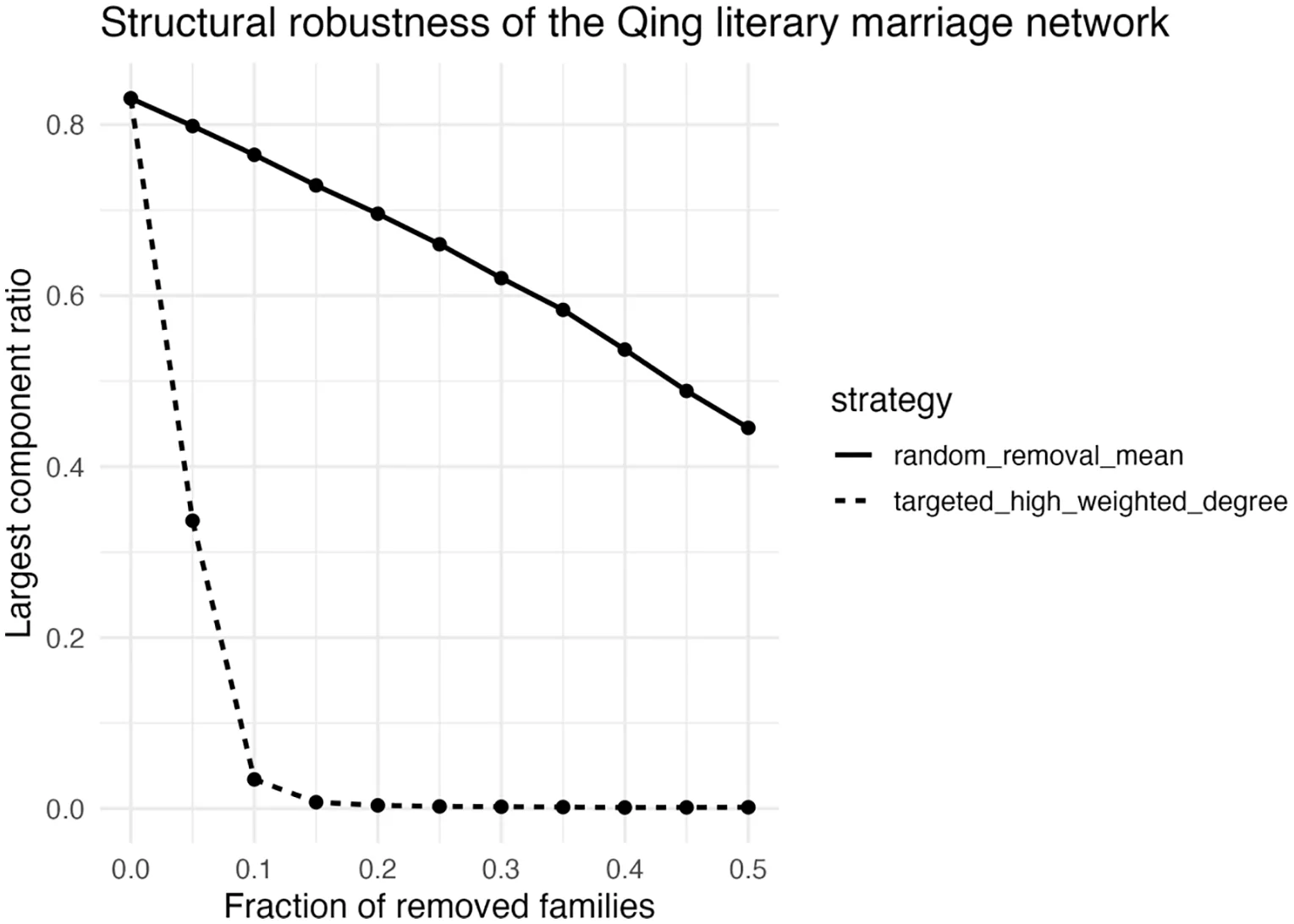
Structural robustness of the Qing literary marriage network.

The figure compares targeted removal of high-weighted-degree family nodes with random node removal. The y-axis reports the proportion of nodes remaining in the largest connected component. The simulation is interpreted as a structural robustness analysis and not as direct evidence of historical adaptation to specific political shocks.

## 5. Discussion

### 5.1. Main findings

This study reconstructed a large-scale marriage network among Qing literary families and examined how network position was associated with recorded literary visibility. The revised analysis adopts a conservative and reproducible surname–location aggregation strategy, producing a cleaned analytical network of 5,197 parseable marriage records, 657 focal literary-family nodes, 2,704 total family nodes, and 3,541 distinct weighted family-pair edges. The results show that the network was large, modular, and regionally clustered, with a large connected component linking most family nodes.

The most consistent statistical finding is that the number of distinct marital partners, measured by unweighted degree, is positively associated with the Literary Capital Proxy. This result suggests that families connected to a broader range of marriage partners tended to have greater recorded literary visibility in the source corpus. This association remains significant after controlling for record-exposure and core-region differences and after using province-clustered robust standard errors.

The revised results also require a more cautious interpretation than the original manuscript. Weighted degree is positively associated with LCP in some specifications, but the association is weaker than that of unweighted degree. Betweenness centrality does not show the expected positive relationship with literary visibility; instead, it is negatively associated with LCP in the exposure-adjusted models. Eigenvector centrality is not statistically significant. These findings indicate that literary visibility was more closely related to the breadth of distinct marital connections than to repeated marriage volume, brokerage position, or connection to already central families.

### 5.2. Marriage breadth and recorded literary visibility

The positive association between unweighted degree and LCP suggests that the range of distinct marital partners may have mattered more than the sheer number of repeated marriage ties. In the context of Qing literary families, a broader marriage range could have increased opportunities for textual circulation, family reputation, educational exchange, and the preservation of women’s literary works across households. Marriages linked not only individuals but also genealogical memory, educational practices, and literary reputation. A family connected to many distinct partner families may therefore have had more channels through which its literary members were recorded, cited, anthologized, or remembered.

This does not mean that marriage breadth caused literary capital accumulation. The association may also reflect reverse selection: families with stronger literary reputations may have attracted more marriage partners. It may also reflect shared background factors, such as wealth, officeholding, regional educational infrastructure, and archival survival. The revised analysis therefore interprets the relationship as an association between marriage-network breadth and recorded literary visibility, not as proof that marriage networks directly produced literary achievement.

The distinction between weighted and unweighted degree is important for this interpretation. Weighted degree captures repeated recorded marriage ties, while unweighted degree captures the number of distinct partner families. The stronger performance of unweighted degree suggests that diversity of marital connections may be more relevant to recorded literary visibility than repeated ties with a smaller group of families. This finding refines the original argument by shifting attention from marital intensity to marital breadth.

### 5.3. Female authorship and the visibility of literary families

The Literary Capital Proxy used in the revised analysis includes confirmed female-writer clues extracted from the parsed source text. This measure is conservative because it only captures cases in which women’s authored works or literary identity could be identified from the OCR-based corpus. Even with this limitation, several highly central families also show strong female-authorship visibility, especially those with recorded women writers whose works circulated through marriage, family memory, or later bibliographic documentation.

Marriage was one of the main institutional pathways through which women moved between lineages. Women writers could carry literary training, poetic practice, manuscript culture, and family memory from their natal families into marital households. Conversely, the preservation of women’s literary names often depended on the combined archival practices of both natal and marital families. The network perspective therefore helps show why women’s literary visibility should not be treated only as an individual achievement. It was also shaped by the relational structure linking families. This interpretation is consistent with studies of women’s literary memory and gendered literary transmission in late imperial China [18,19].

At the same time, the revised analysis does not claim that female authorship was fully captured by the available data. The LCP is based on observable textual clues and is likely to undercount women whose works were lost, unpublished, unattributed, or not recorded in the source materials. The results should therefore be understood as evidence of recorded female literary visibility rather than a complete measure of women’s literary production in Qing society.

### 5.4. Reconsidering brokerage and centrality

The negative coefficient for betweenness centrality in the revised models is one of the most important changes from the original interpretation. The original manuscript expected brokerage position to be positively associated with literary capital, assuming that families bridging otherwise separate clusters would be more likely to accumulate cultural resources. The revised results do not support this assumption.

Several explanations are possible. Families with high betweenness may have connected geographically or socially distant groups without necessarily having dense literary records of their own. In a modular historical network, brokerage may reflect structural location rather than cultural prominence. Some bridge families may have been important for linking clusters but less visible in literary catalogues or women’s authorship records. In addition, betweenness is sensitive to network topology and may identify families that serve as connectors between communities rather than families with the strongest literary reputation.

This finding does not weaken the value of network analysis. Instead, it shows that different centrality measures capture different social mechanisms. Unweighted degree reflects the breadth of direct marital connections, weighted degree reflects repeated marital volume, betweenness reflects bridging position, and eigenvector centrality reflects proximity to central families. The revised analysis indicates that these measures should not be interpreted interchangeably. In this dataset, breadth of direct partner families is more consistently related to recorded literary visibility than brokerage or prestige-based centrality.

### 5.5. Structural robustness rather than historical causality

The network has a large connected component and high modularity, indicating that Qing literary marriage relations combined broad connectivity with strong clustering. These features may be discussed in a limited network-topological sense of structural robustness, rather than as full evidence of social-ecological resilience [20,21]. A modular network can contain dense local clusters while still maintaining cross-cluster connections through selected families.

However, the revised study does not treat this analysis as proof of historical adaptation to specific shocks. The available data do not provide sufficiently precise temporal ordering to establish how the network changed before, during, or after events such as the Taiping Rebellion. The results therefore support only a structural interpretation: the marriage network displayed modular and partially connected features that may have shaped the circulation and preservation of literary visibility across families.

This clarification is important because resilience claims require either longitudinal evidence, counterfactual comparison, or explicit event-based analysis. Without such evidence, the more appropriate conclusion is that the marriage network displayed a modular and partially connected structure that may have shaped the circulation and preservation of literary visibility across families.

### 5.6. Cultural sustainability and historical interpretation

The concept of cultural sustainability is useful for interpreting how literary memory, textual practices, and family reputation persisted across generations. In the Qing context, cultural continuity did not depend only on individual authors or isolated households. It was also embedded in kinship, marriage, regional education, and inter-family recognition. The reconstructed network shows one possible relational structure through which literary visibility was associated with family alliances.

The revised findings support a moderate version of the cultural-sustainability argument. Families with a wider range of direct marriage partners tended to have higher recorded literary visibility, suggesting that marriage networks may have contributed to the circulation and preservation of cultural resources. Yet the evidence does not justify a strong claim that marriage networks caused cultural sustainability. Cultural transmission was likely shaped by multiple factors, including examination success, wealth, publishing opportunities, lineage organization, women’s education, local scholarly communities, and the survival of archival records.

Therefore, the contribution of this study lies not in proving a single causal mechanism, but in demonstrating how historical network reconstruction can make kinship-based cultural transmission more visible and analytically testable. By combining source-based reconstruction with network measures and robustness checks, the study provides a reproducible framework for examining the relational foundations of literary visibility in late imperial China.

### 5.7. Limitations

Several limitations should be acknowledged. The first limitation concerns data reconstruction. Xu’s printed source records 5,289 marriage entries involving 766 literary-family entries, but the reproducible OCR-based analytical dataset contains 5,197 parseable records and 657 surname–location focal family nodes after normalization. This difference reflects damaged OCR markers, irregular punctuation, repeated headings, and the conservative aggregation of homonymous families from the same locality. The revised dataset is therefore designed for transparency and reproducibility rather than exhaustive recovery of every genealogical distinction.

The second limitation concerns the Literary Capital Proxy. The revised analysis does not use a full examination-success component because complete jinshi and juren counts could not be reconstructed reliably from the OCR corpus. The LCP therefore captures observable literary-output and female-authorship clues rather than total cultural capital. Families with substantial literary achievement but weak textual visibility in the parsed source may be underestimated.

The third limitation concerns causality. The network is reconstructed primarily from historical marriage records and does not provide sufficient temporal precision to establish whether marriage ties preceded literary visibility. The observed associations may reflect reverse selection, omitted variables, or common regional conditions. Wealth, officeholding, education, local publishing culture, lineage organization, and archival survival may all have influenced both marriage opportunities and literary visibility.

The fourth limitation concerns regional clustering. Although the revised models use province-clustered robust standard errors and core-region controls, province-level adjustment cannot fully account for local variation within Jiangnan or for differences in source preservation across counties and lineages. Future research could improve this analysis by integrating county-level coordinates, officeholding records, examination registers, and manually verified family-level literary catalogues.

### 5.8. Future research

Future studies can extend this analysis in several directions. A manually verified dataset linking each family to examination success, officeholding, printed works, women’s writings, and county-level location would allow a more complete reconstruction of literary capital. More precise temporal coding would make it possible to test whether marriage ties preceded literary visibility and whether network structure changed around major historical events. Spatial models could further evaluate whether regional dependence shapes the observed relationship between marriage networks and literary visibility.

Another promising direction is comparative analysis. Qing literary families can be compared with merchant lineages, official families, local gentry groups, or women’s literary communities to determine whether the association between marital breadth and cultural visibility was specific to literary lineages or reflected broader elite strategies. Such comparisons would help clarify the role of marriage networks in the long-term preservation and circulation of cultural resources.

## 6. Conclusion

This study reconstructed and analyzed a large-scale marriage network among Qing literary families using a revised and reproducible surname–location aggregation strategy. Based on 5,197 parseable marriage records, the final analytical network contains 657 focal literary-family nodes, 2,704 total family nodes, and 3,541 distinct weighted family-pair edges. The network is characterized by a large connected component, strong modularity, and a structure in which focal literary families were linked not only to one another but also to a wider set of non-focal marriage partners.

The revised findings show that the breadth of direct marital connections is associated with recorded literary visibility. In particular, unweighted degree, measured as the number of distinct partner families, remains positively associated with the Literary Capital Proxy after controlling for record exposure, regional differences, and province-level clustering. This suggests that families connected to a broader range of marital partners tended to have higher visibility in the available literary and female-authorship records.

At the same time, the revised analysis does not support a simple centrality-based explanation of literary visibility. Betweenness centrality is negatively associated with the Literary Capital Proxy in the exposure-adjusted models, and eigenvector centrality is not statistically significant. These results indicate that brokerage position and connection to highly central families should not be automatically interpreted as evidence of greater literary capital. Different centrality measures capture different aspects of network position, and in this dataset the most stable association is with the diversity of direct marriage partners.

The study also clarifies the role of women’s literary visibility within family networks. Confirmed female-writer clues contribute to the Literary Capital Proxy and show that women’s literary presence was embedded in inter-family marriage relations. However, because the proxy relies on observable textual clues in the parsed corpus, it should be interpreted as a conservative measure of recorded literary visibility rather than a complete reconstruction of Qing women’s literary production.

Overall, this study provides a reproducible network-based framework for examining how kinship and marriage were associated with the preservation, circulation, and intergenerational transmission of literary visibility in Qing China. In this limited historical sense, the findings help clarify how cultural sustainability can be studied through observable relational structures rather than treated as an abstract concept. The results support a cautious interpretation: marriage networks were associated with cultural transmission, but the available evidence does not establish a direct causal effect of marriage ties on literary capital accumulation or historical adaptation to specific shocks. Future research can improve on this study by integrating manually verified examination records, officeholding data, county-level spatial information, and more precise temporal coding.

## References

[1] Elman BA. A Cultural History of Civil Examinations in Late Imperial China. Berkeley, CA, USA: University of California Press. 2000.

[2] Chow KW. Publishing, culture, and power in early modern China. Stanford, CA, USA: Stanford University Press. 2004.

[3] Pan G. Eminent Clans of Jiaxing in the Ming and Qing Dynasties. Shanghai, China: Shangwu Yinshuguan. 1937.

[4] Mann S. The Talented Women of the Zhang Family. Berkeley, CA, USA: University of California Press. 2007.

[5] Xu Y. 清代文学世家姻亲谱系. Nanjing, China: Fenghuang Chubanshe. 2010.

[6] Bourdieu P. The forms of capital. In: Richardson J, editor. Handbook of Theory and Research for the Sociology of Education. New York, NY, USA: Greenwood. 1986. p. 241–58.

[7] Bourdieu P, Passeron JC. Reproduction in Education, Society and Culture. 2nd ed. London, UK: Sage. 1990.

[8] Beattie HJ. Land and lineage in China: A study of T’ung‑ch’eng County, Anhwei, in the Ming and Ch’ing dynasties. Cambridge, UK: Cambridge University Press. 1979.

[9] Tsui LH, Wang H. Harvesting Big Biographical Data for Chinese History: The China Biographical Database (CBDB). J Chin Hist. 2020;4(2):505–11. doi: 10.1017/jch.2020.21

[10] Ho HI. Distant Reading East Asian Literary History: New Methods and Case Studies. Leiden, The Netherlands: Brill. 2021.

[11] Tsai RT-H, Liu Y-S, Chen Y-J, Hsieh HY, Chan Y-C. Digital humanities approach to analyzing the roles and military power of Supreme Commanders and Grand Coordinators in the Ming Dynasty: a computational analysis of Ming Shilu. Digital Scholarship in the Humanities. 2025;40(3):910–27. doi: 10.1093/llc/fqaf024

[12] Huang S-Y, Wu S-Y, Chen Y-J, Tsai RT-H, Fan I-C. Climate event classification based on historical meteorological records and its presentation on a Spatio-Temporal research platform. Digital Scholarship in the Humanities. 2022;37(4):1022–32. doi: 10.1093/llc/fqab099

[13] Padgett JF, Ansell CK. Robust action and the rise of the Medici, 1400–1434. Am J Sociol. 1993;98(6):1259–319.

[14] Adams J. The familial state: Ruling families and merchant capitalism in early modern Europe. Ithaca, NY, USA: Cornell University Press. 2005.

[15] Wasserman S, Faust K. Social Network Analysis: Methods and Applications. Cambridge University Press: Cambridge, UK, 1994.

[16] Dewald J. The formation of a provincial nobility: The magistrates of the parlement of Rouen, 1499–1610. Princeton, NJ, USA: Princeton University Press; 1980.

[17] Blondel VD, Guillaume J-L, Lambiotte R, Lefebvre E. Fast unfolding of communities in large networks. J Stat Mech. 2008;2008(10):P10008. doi: 10.1088/1742-5468/2008/10/p10008

[18] Fong GS. Inscribing a sense of self in mother’s family: Hong Liangji’s memoir and poetry of remembrance. Nan Nü. 2008;10:27–61.

[19] Zhang H. Gender and Literary Studies in the Ming and Qing Dynasties. Nanjing, China: Jiangsu Guji Chubanshe. 2010.

[20] Folke C. Resilience: The emergence of a perspective for social–ecological systems analyses. Glob Environ Chang. 2006;16:253–67.

[21] Folke C, Biggs R, Norström AV, Reyers B, Rockström J. Social-ecological resilience and biosphere-based sustainability science. Ecol Soc. 2016;21:41.

